# Spatially resolved fibre cavity ring down spectroscopy

**DOI:** 10.1038/s41598-020-76721-y

**Published:** 2020-11-19

**Authors:** Rongzhang Chen, Zhaoqiang Peng, Mohan Wang, Aidong Yan, Shuo Li, Sheng Huang, Ming-Jun Li, Kevin P. Chen

**Affiliations:** 1 Department of Electrical and Computer Engineering, University of Pittsburgh, Pittsburgh, PA 15213 USA; 2 Corning Incorporated, One Riverfront Plaza, Corning, NY 14831 USA

**Keywords:** Electrical and electronic engineering, Applied optics, Optical techniques

## Abstract

This paper presents a fibre cavity ring down spectroscopy probed by Rayleigh scattering optical frequency domain reflectometry (OFDR), which provides spatial location of stimuli and improved signal to noise ratio for distributed sensing measurements. A section of optical fibre was integrated into an active fibre ring cavity with optical gain and interrogated by the OFDR system for 11 cycles with a single laser scan. Through the cavity ring down configuration, root-mean-squared (RMS) noise of distributed temperature and strain measurements was reduced to 6.9 mK and less than 0.1 με, respectively for 1-cm spatially resolved measurements. Our work shows that the active fibre cavity configuration can be combined with distributed fibre sensing schemes to achieve both high spatial resolution and high sensitivity measurements.

## Introduction and background

Cavity ring-down spectroscopy (CRDS) is one of the most widely used optical sensing techniques for ultra-high sensitivity measurements^1^. A conventional CRDS measurement is performed in the time domain. Laser pulses, from a tuneable laser, are injected into a high finesse cavity, and stimuli induced minuscule optical loss can be accumulated and amplified through repeated cycling of optical pulses in the cavity. This leads to drastic improvements in detection sensitivity and reliability. Since its inception, various versions of CRDS have been constructed for use in challenging applications with need for physical and chemical sensing. However, the biggest drawback of the current CRDS system is the lack of spatial information. Since the measurement is performed in the time domain, using laser pulse with pulse duration in nanoseconds, it is exceedingly difficult to pin-point the exact location in space where optical loss or index change occurs.

Recently, a number of research groups have suggested that optical fibre based CRDS (FCRDS) can be used to increase cavity length to enhance measurement sensitivity and reduce the technical difficulties in optical alignments^2^. In previous studies on FCRDS, measurements were carried out with using pulsed probing light in the time domain, which only measured cumulative effects, as its free-space counterparts. In this report, we describe a FCRDS technique using continuous waves (CW)^3^ in the spatial domain. FCRDS has been successfully used to measure weak strain^4^, temperature^5^, and in-fibre optical loss^6^. In fact, the foremost potential of the FCRDS scheme is the ability to perform spatially resolved CRDS thanks to the various existing distributed fibre sensing schemes such as Rayleigh backscattering optical frequency domain reflectometry (OFDR)^7^ and Brillouin backscattering optical time domain reflectometry (OTDR)^8^.

In this report, we demonstrate a spatially resolved FCRDS technique enabled by the Rayleigh OFDR scheme where the location of physical stimuli can be measured with both great accuracy and high spatial resolution. This technique utilizes the OFDR method to interrogate distributed Rayleigh signal along optical fibre with tens of micrometre-level spatial resolution^7^. By monitoring the spectral shift of backscattered Rayleigh signals, distributed strain and temperatures changes can be interrogated with improved sensitivity and repeatability. As a useful distributed sensing technique, Rayleigh OFDR schemes have been used to perform distributed loss, temperature, and strain measurements in a variety of areas that utilize physical sensing^9–15^ including structural health monitoring^16,17^, process control^18,19^, and security/safety monitoring^20^.

Since the inception of Rayleigh OFDR, continuous efforts have been made to improve the performance of this distributed sensing scheme. The currently available and commercial Rayleigh OFDR system has a temperature and strain measurement accuracy of ± 0.1 °C and ± 1 µε respectively^21^. This is largely limited by several factors, including: the cross-correlation noise of random Rayleigh backscatter signals in the spectral domain^7^, ambient temperature fluctuation along the fibre, and detector intensity noises in the system^22^. The cross-correlation noise comes from the intrinsic properties of the optical fibre, however, this comes with variability as the spectrum of a section of optical fibre is arbitrary and as a result, any relative shifts of the optical spectra deduced from cross-correlation results are subject to uncertainty. At short gauge length *Δx* (i.e. several milimeters), the measurement noises are dominated by cross-correlation uncertainty, which can be qualitatively described as^7^,

1 $$ \begin{gathered} \Delta x\varepsilon_{res} = \frac{\lambda }{4n} \hfill \\ \Delta xT_{res} = \frac{\lambda }{4n}\left( {\frac{d\varepsilon }{{dT}}} \right)^{ - 1} , \hfill \\ \end{gathered} $$

where *ε*_*res*_ and *T*_*res*_ correspond to strain and temperature resolutions respectively, *λ* is the average probing wavelength, *n* is equal to the refractive index of the optical fibre, Δ*x* is the gauge length of the interrogation. As the gauge length becomes larger, the other two noise components become major factors. UV exposure has been demonstrated to successfully reduce measurement noise by an order of magnitude in gauge lengths of centimetres by reducing photodetector detection noises^22^. In this scheme, both UV laser exposure and hydrogenation treatments on optical fibres are required to raise the background of Rayleigh backscattering signals.

Here, we provide another method to reduce measurement associated noise that can be implemented for use in all fibres without further fibre handling and processing. The noise acquired during measurement can be significantly reduced using FCRDS configuration, which preserves all the benefits of distributed measurements. Using the experimental setup described here, the OFDR system is able to interrogate the same section of optical fibre repeatedly with one laser scan, which suppresses random noises and increases the measurement accuracy. Further, since the FCRDS measurement presented here is performed in the spatial domain, this technique can spatially resolve where the stimuli occur with high spatial resolution (1 cm).

## Method

**Figure 1:**
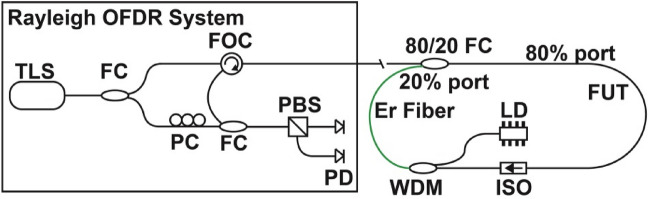
Schematic of the spatially resolved FCRDS. *TLS* tuneable laser source, *F*C fibre coupler, *PC* polarization controller, *FOC* fibre optic circulator, *PBS* polarizing beam splitter, *FUT* fibre under test, *PD* photodetector, *LD* laser diode, *ISO* isolator, *WDM* wavelength division multiplexer.

A schematic of the experimental setup is shown in Fig. 1. The experiment used a commercially available Rayleigh OFDR system (OBR4600 from Luna Innovations) as the interrogation instrument. During the experiment, the scanning range of the tuneable laser in the OFDR system was set as 43.09 nm centred at 1567 nm. Thus a spatial resolution of 20 µm and an interrogation length up to 70 m were configured to interrogate the fibre cavity. An 80/20 fibre coupler (FC) was used as a light combining item, where the CW laser emission from the OFDR unit was injected into the cavity via the 80% port with an average loss of ~ 1.1 dB per pass. Then a section of ~ 3-m-long single mode fibre (SMF-28, Corning) was used as the fibre under test (FUT). After the FUT, an isolator (ISO) was used to force light to propagate clockwise only inside the optical cavity. The ISO introduced an additional ~ 0.6 dB loss to the optical cavity but prevented any unwanted rings of backscattered Rayleigh signals.

With the ISO and FC only to form the cavity, the net loss between each ring-down cycle except the first, were 8.3 dB measured by the OFDR system. This included the loss of ~ 7.0 dB at 20% port of FC when the probing light exited the current cycle to start a new one and ~ 1 dB loss at 80% port as the backscattered signal propagated back to the interrogator. Given that the OFDR system has about a 20-dB dynamic range above the noise floor, up to 3 cycles of backscattered signals from the ring-down processes can be measured. Distributed measurements inside the fibre loop can also suffer from poor signal to noise ratio (SNR) at the photodetectors due to excessive optical loss at the last cycle. Therefore, an in-cavity Er-doped fibre amplifier (EDFA) was necessary and was set up after the ISO^23^ to compensate for major optical loss of the probing light that occurred at the 20% port of FC. The gain provided by the EDFA expanded the number of measurable ring-down cycles to over 11 with significantly better SNR. As shown in Fig. 1, the EDFA consists of a 980-nm laser diode (JDSU S27-7602-460) with 120-mw pumping power, a WDM and a 3-m-long Erbium doped optical fibre (Corning Er-1600 L3), configured in a forward pumping arrangement. As the probing light rings down the cavity, the Rayleigh backscattered signal produced by the FUT at different ring-down cycles were measured sequentially in spatial domain.

## Results

**Figure 2:**
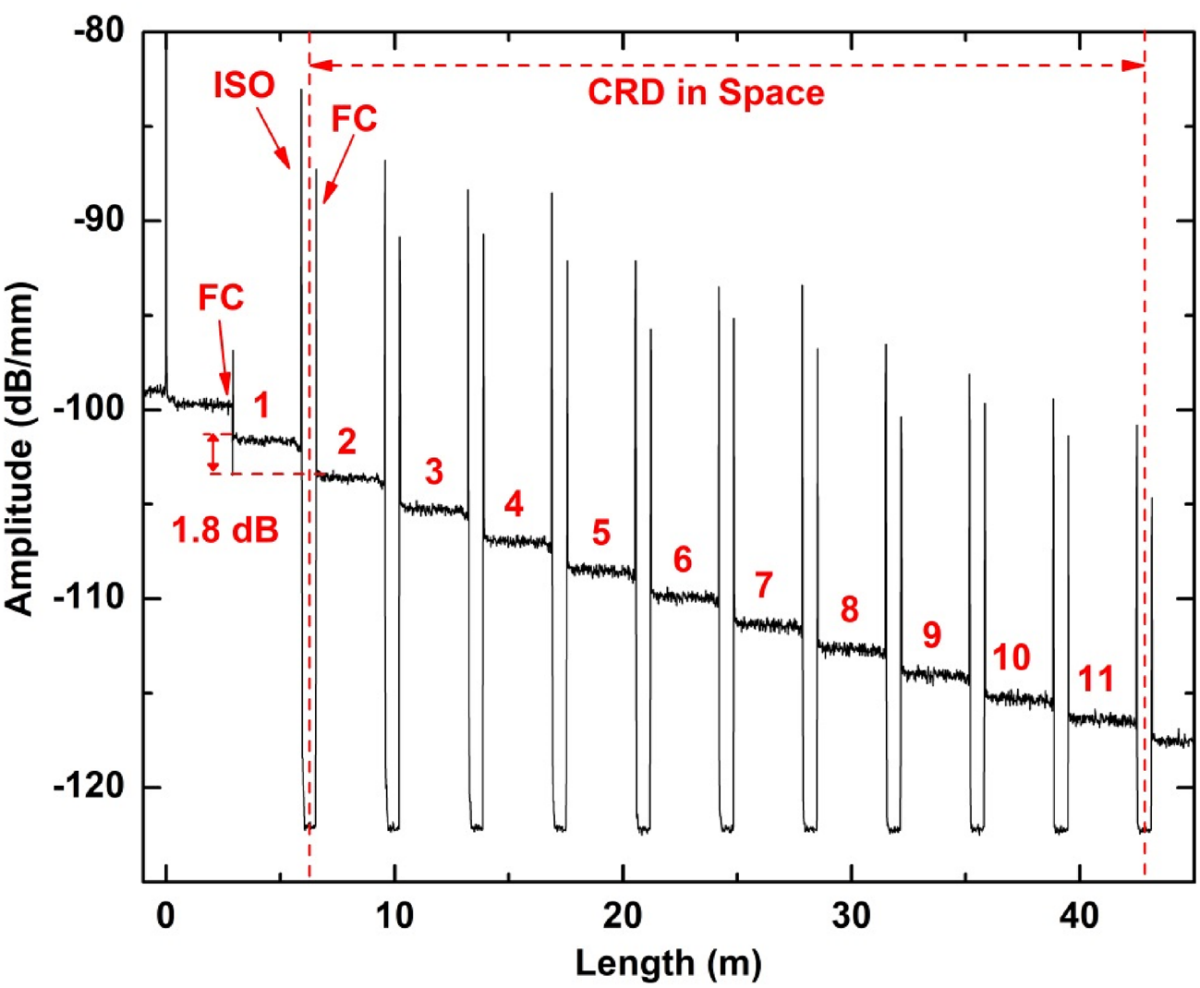
Typical CRD decay transient response measured in spatial domain using the Rayleigh OFDR system. *FC* fibre coupler; *ISO* isolator.

A typical cavity ring down (CRD) decay transient response measured by the OFDR unit in the spatial domain is demonstrated in Fig. 2. We used 1 to 11 to designate cycles measured by the OFDR system on the same section of the FUT. Optical fibre and EDFA components after the ISO did not return any Rayleigh backscattering, so they corresponded to a steep signal drop in the OFDR measurements at the end of each ring-down cycle.

After each ring-down cycle, a net amplitude drop of Rayleigh signal still existed as shown in Fig. 2. This is because EDFA cannot fully compensate the loss in each cycle but only reduces it from 8.3 to 1.8 dB/cycle. Due to this loss of residual power, 11 ring-down cycles were acquired for further spatial-resolved temperature and strain measurements. Ring-down measurements beyond the 11th cycle were not analysed due to the high SNR of the photodetector, where signals are less than 5 dB above the instrument’s noise floor. An intensity detection with very low SNR for cycles beyond 11 can transition to high noises in strain/temperature measurement. Including those very noisy measurements will defeat the purposes of noise suppression for the proposed method.

It is possible that a more elaborate^24^ intra-cavity setting could reduce the per-cycle optical loss to well less than 1-dB, which will extend the usable number of ring-down cycling farther. However, at a sensing gauge length of 1 cm, the max measurement range of the OFDR is at 70 m. Therefore, the current 11-cycle measurement has already probed most of the measurement range for ~ 50 m as shown in Fig. 2. However, using an OFDR instrument with extended measurement range, it would be beneficial to increase the spatial-resolved ring-down cycles for better measurement accuracy. It is also worth noting that the EDFA system in this setup has induced intra-cavity laser operation at 1562 nm. The coexisting lasing components combined with a non-uniform gain profile of the EDFA might modify the original intensity profile of the scanning laser of the OFDR. However, our experiment results indicate that no degradation in measurement is observed. This is probably because the OFDR method carries out sensing by measuring the spectral shift induced by perturbation under test. Such a procedure does not require a uniform laser intensity profile.

Several pairs of reflection spikes that separate different ring-down cycles can be observed in Fig. 2. Each pair comes from the internal interface reflection of ISO and FC sequentially. The labelled ISO reflection occurs as probing light first enters the cavity and gets reflected by internal components of ISO; while the labelled FC reflection is derived from the internal reflection of FC when amplified probing light passes it via its 20% port, prior to entering the 2nd ring-down cycle. OFDR measurement in Fig. 2 yields a distance of ~ 94 cm between any pair of reflection spikes, which does not correspond to the physical length of the EDFA as ISO prevents OFDR unit from receiving any backscatter signals past it. It is worth noting that the strong reflection spikes affect sensory measurement of the neighboring fibre sections because their side lopes are usually strong enough to shadow any real signals. Therefore, about 30-cm-long sections of fibres adjacent to those spikes were excluded from our data analysis.

### Temperature measurement

The spatially resolved FCRDS was first performed for distributed temperature measurements to study the root mean square (RMS) fluctuation of the measurement along the fibre. A 1.6-m long section at the middle of the FUT as shown in Fig. 1 was stripped of its plastic jackets and placed in a thermal-isolated enclosure where ambient temperature fluctuation was minimized. OFDR distributed measurement always measures strain/temperature changes with respect to a pre-defined initial condition. Therefore, with proper thermal isolation and no induced temperature changes between initial and test conditions, the distributed temperature measurements should return all zero temperature reading and RMS. The same section of the optical fibre was used to perform comparisons in two scenarios, the spatially resolved FCRDS and the normal OFDR measurements. Cutting and re-splicing of the optical fibres were conducted for switching between tests. Given the thermal isolation enclosure and relatively short duration of measurement times, ~ 10 s, the sensed temperature fluctuation along the fibre can be attributed to photodetector noise and the cross-correlation noise only.

**Figure 3:**
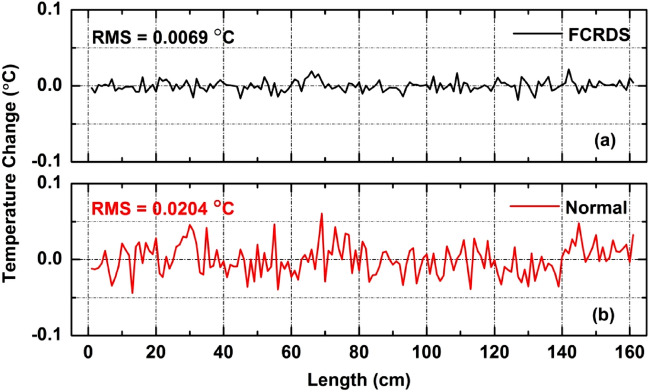
Comparison of temperature measurement noise levels of (**a**) spatially resolved FCRDS and (**b**) normal OFDR method.

Experiment results are shown in Fig. 3 with the gauge length of 1-cm. Using the normal OFDR interrogation, a distributed measurement on 1.6-m long FUT section is shown in Fig. 3b, where RMS noise along the FUT was measured as 0.0204 °C. As a comparison, the distributed temperature measurement using the spatially resolved FCRDS is shown in Fig. 3a. A total of 11 ring-down cycles of temperature measurements were performed in a single laser scan. And all the measured temperature profiles of the FUT were averaged to result in a final temperature profile, plotted in Fig. 3a. Similar to a conventional FCRDS, which achieves high measurement accuracy and sensitivity through signal accumulation over repeated measurements, the presented FCRDS averages repeated measurements to reduce noise. The proposed FCRDS measurement yielded a temperature RMS fluctuation of 0.0069 °C, and 66.2% reduction from the normal method.

**Figure 4:**
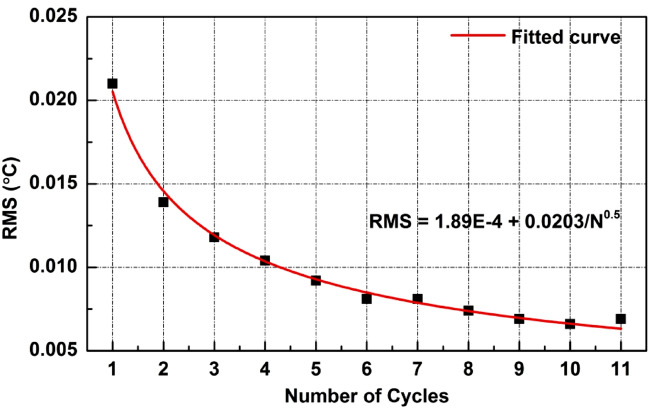
RMS noise level of the distributed temperature profile versus number of ring-down cycles considered. A curve in red is provided to fit the experimental results.

Figure 4 reveals how the spatially resolved FCRDS reduces the measurement noise by repeated measurements. The accumulated effect of the noise reduction was achieved by averaging each cycle of measurements obtained in the ring-down process. As shown in Fig. 4, the RMS noise level over the FUT decreases as more repeated measurements were factored in. With a maximum of 11 measurements in one ring-down process, the theoretic reduction rate for random noises is equal to $1/\sqrt{N}$ = 69.9%, where *N* is 11. Our experiment results presented here match well with this estimation. It is evidenced by the curve fitting in Fig. 4, where the rate of RMS noise reduction is dominated by the factor of $1/\sqrt{N}$. This result indicates that the proposed FCRDS method is able to suppress the random photodetector noises in the OFDR system and the cross-correlation noises at this gauge length are not dominant.

**Figure 5:**
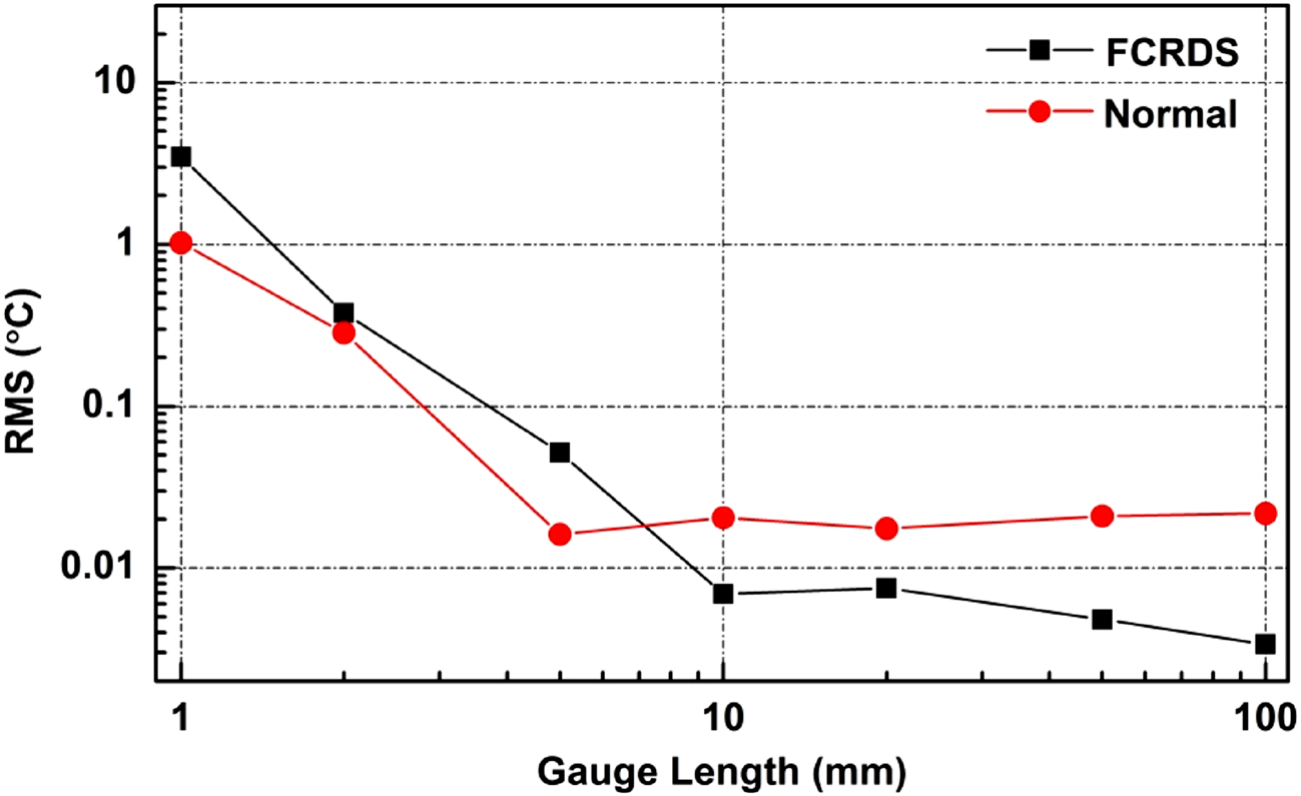
Temperature measurement noises as a function of gauge length with the two sensing methods. *FCRDS* spatially resolved FCRDS, *Normal* normal OFDR measurement.

To study the noise reduction at other gauge lengths, gauge lengths between 1 mm to 1 m were tested. For consistency, 11-fold of acquired measurements were always used for averaging across all gauge length settings. The results are shown in Fig. 5. With a gauge length of < 5 mm, the noise of the OFDR system is mainly governed by the theoretic uncertainty of Rayleigh scatter^22^. Noise suppression via repeated measurements and averaging cannot be expected for this noise as the cross-correlation uncertainty is not a random process that is consistent for any specific section of the fibre. Moreover, additional measurement noises occur in far ring-down cycles as more photo detector noises are prone to happen in distant cycles and the small gauge length setting does not allow enough data points to average them out. Those additional measurement noises from the distant (far) cycles leaked into the RMS noise level of the proposed method via averaging. This explains why with gauge lengths that were ≤ 5 mm the proposed method had higher noise RMS. However, benefits of the proposed method become increasingly clear as the gauge length is ≥ 10 mm where the cross-correlational uncertainty becomes less important and photodetector noises start to dominate. The increased gauge length also allows more data points in each gauged section for integration which improves sensory SNR for the far or distant cycles. With these two effects combined, the true noise reduction is revealed. As shown in Fig. 5, the proposed method continuously drives down the RMS noise level. At 100-mm gauge length, the maximum percentage of noise reduction is at 84.3%.

### Strain measurement

**Figure 6:**
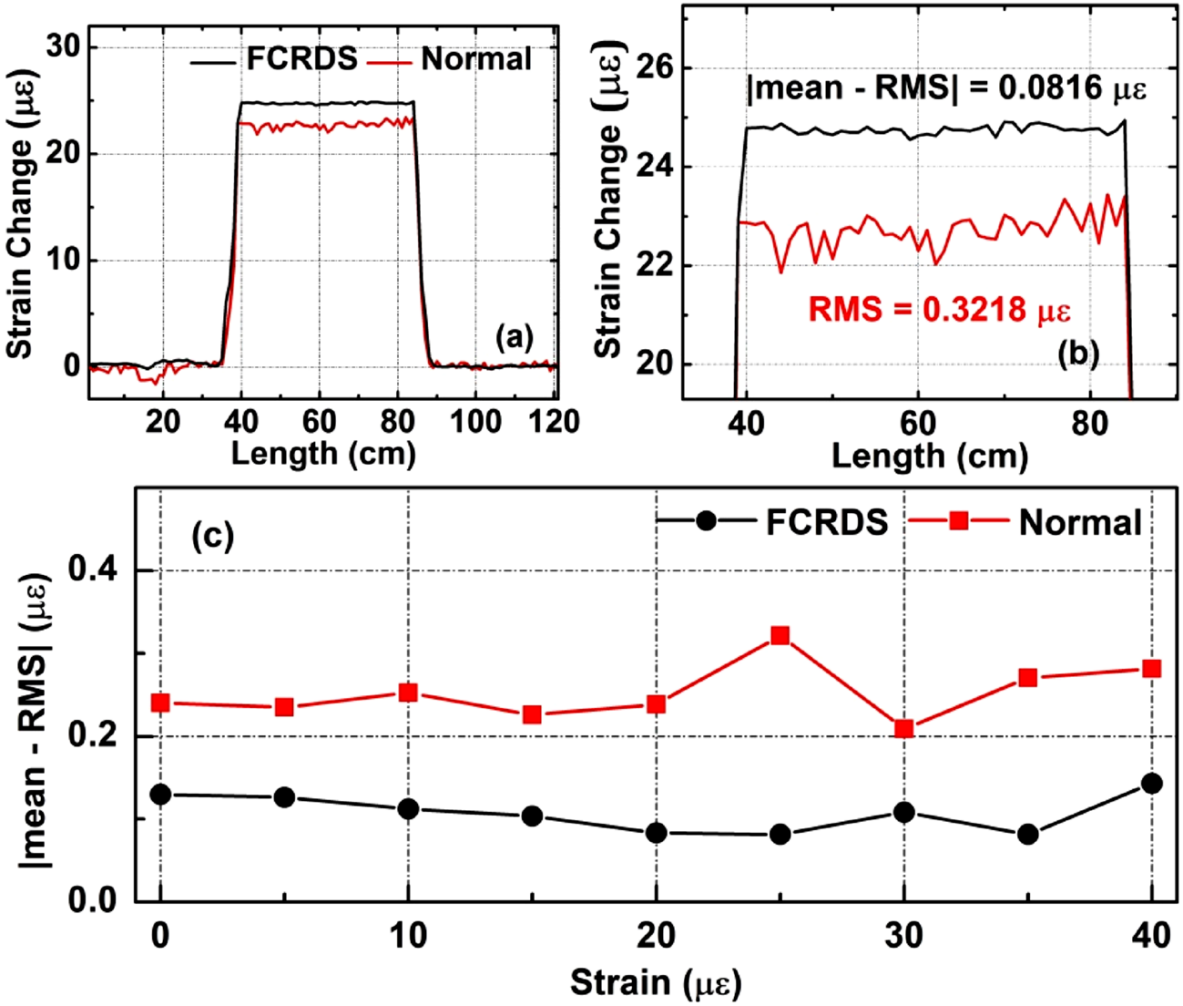
Measured strain change profiles at (**a**) 25 µε, (**b**) a local zoom-in of (**a**), and (**c**) a summary of |mean − RMS| noise levels at all tested strain changes.

To further characterize the spatial resolved FCRDS technique, distributed strain measurements were carried out in a fibre ring-down cavity. The gauge length of the OFDR system was set at 1-cm. The optical fibre for stretching is a section of 45-cm-long stripped optical fibre at the middle of the FUT that was previously employed in Fig. 1. Both sides of the optical fibre used for stretching were glued to metal fixtures with epoxy. A linear stage (Newport, M-UTM100CC1DD) was used to stretch the fibre from one end of the fixture to the other. In a reference strain test, the same FUT section was cut and re-spliced to fit a normal OFDR measurement. To reduce ambient perturbation from air flow and thermal fluctuation, the entirety of the stretched area of the FUT was covered with a cardboard enclosure. As an initial condition, the optical fibre was pre-strained by 110 µε before any tests were conducted. In Fig. 6a, two experimental results of spatially resolved strained measurements of the fibre are compared. In both scenarios, one laser scan of the instrument was carried out while the spatially resolved FCRDS method was able to provide 11-fold of the distributed measurements. The optical fibre section was stretched with additional 25 µε for the actual change in strain measurement. Figure 6a highlights the full advantage of the proposed spatially resolved FCRDS, where not only the location of stimuli (strain) applied to the fibre could be determined with 1-cm spatial resolution but also noise measurement was suppressed. Figure 6b details the RMS noise reduction on the section of stretched fibre. The ring-down cavity yielded 64.9% reduction in RMS noises. The slight difference in strain levels for the two measurements in Fig. 6a,b was due to insufficient reproducibility of the linear motion stage across the two strain tests. Additional strain measurements were conducted and presented in Fig. 6c where the optical fibre was stretched between 5 and 40 µε. Overall, the spatially resolved FCRDS yielded consistent noise reduction with an average of 56.5% in distributed strain measurements. The fluctuation of noise suppression rate could be observed, probably due to the instability of the motion stage and residual ambient temperature perturbation among the different tests.

## Discussions and conclusions

This paper explores technical opportunities arise from nexus of cavity ringdown spectroscopy and distributed fibre sensing technology. Using the OFDR interrogation scheme, the spatial information of stimuli in a fibre cavity can be preserved during the ringdown measurement and can be determined with 1-cm spatial resolution. This paper demonstrates that if there is a local refractive index change caused by strain or temperature, the OFDR-enabled FCRDS technique can average measurements of this local change from each cycle to significantly improve the accuracy and spatial resolution. Using the same technique, the OFDR technology can also accumulate this local optical loss to reach better accuracy, and spatially resolve it. On the other hand, applications of CRDS techniques can also enhance measurement sensitivity and SNR of distributed fibre sensors. In this paper, the introduction of FCRDS into Rayleigh backscattering distributed fibre sensor technique yielded 7 times enhancement of SNR in temperature measurements at the expense of shortening effective interrogation distance. The sacrifice of interrogation length could be a drawback for distributed fibre sensors using an OFDR interrogation scheme. However, introduction of CRDS for measurement performance enhancement could significantly out-weight the concern of lost interrogation length for other distributed sensor interrogation schemes. For example, for Optical Time Domain Reflectometry for Brillouin scattering based distributed sensors, while interrogation length can exceed 100 km, an implementation of a CRDS configuration could significantly improve sensor performance in term of measurement sensitivity. In theory, the OFDR-enabled FCRDS technique discussed in this paper can be achieved through averaging multiple OFDR measurements on the same FUT. However, it is important to note that Rayleigh based distributed sensing is inherently sensitive to environmental disturbance including vibration. Averaging over separated measurements, even when they were taken within a short time span cannot produce improvement measurement accuracy presented in this paper^25^. In addition, the OFDR instrument used in this work can manage repeated measurement but only at ~ 0.1 Hz^21^. This is because that most of tunable diode laser cannot be tuned continuously at high speed while preserving its narrow linewidth.

Results presented in this paper shows that active fibre cavity configuration can be combined with distributed fibre sensing schemes to achieve both high spatial resolution and high sensitivity measurements.
